# Infective Endocarditis Secondary to Mycoplasma pneumoniae

**DOI:** 10.7759/cureus.17461

**Published:** 2021-08-26

**Authors:** Huzaifa Dawood, Saad Nasir, Reem M Khair, Mustafa Dawood

**Affiliations:** 1 Medicine, Naas General Hospital, Naas, IRL; 2 Internal Medicine, Aga Khan University Hospital, Karachi, PAK; 3 Internal Medicine, Beaumont Hospital, Dublin, IRL; 4 Nephrology, Emory University School of Medicine, Atlanta, USA

**Keywords:** infective endocarditis, mycoplasma pneumoniae, infection, case, gram positive bacteria

## Abstract

*Mycoplasma pneumoniae *(MP) is a gram-positive bacterium most commonly associated with community-acquired pneumonia in adults. It can also involve other systems of the body. Cardiovascular complications include pericarditis, myocarditis, congestive cardiac failure, and, rarely, infective endocarditis. We report a case of infective endocarditis secondary to MP infection in an adult. We treated our patient with doxycycline, which showed significant improvement.

## Introduction

*Mycoplasma pneumoniae* (MP) is a gram-positive bacterium responsible for respiratory tract infection in children and young adults and causes mild to severe upper and lower respiratory tract infections termed atypical pneumonia [1]. Reports suggest an incidence of 10-40% of community-acquired pneumonia in some communities [2]. Hepatic, neurological, cardiac, and skin manifestations of MP are well recognized [1]. Other known associated diseases with MP include Steven-Johnson syndrome, toxic epidermal necrolysis, and erythema multiforme [3]. Limited reports in the literature exist of an association of MP with infective endocarditis; however, patients described earlier have a history of associated heart disease [4-6]. Here, we discuss the case of a lady who presented with fever, later diagnosed as infective endocarditis secondary to MP infection without a preexisting heart disease in an immunocompetent individual.

## Case presentation

A 29 -year-old lady with no previous medical history presented to the emergency department after developing a sudden-onset fever, myalgia, and weight loss for the past one month. Her history was negative for any congenital or acquired heart disease, or any recent dental procedures. She had no history of any intravenous drug abuse.

**Figure 1:**
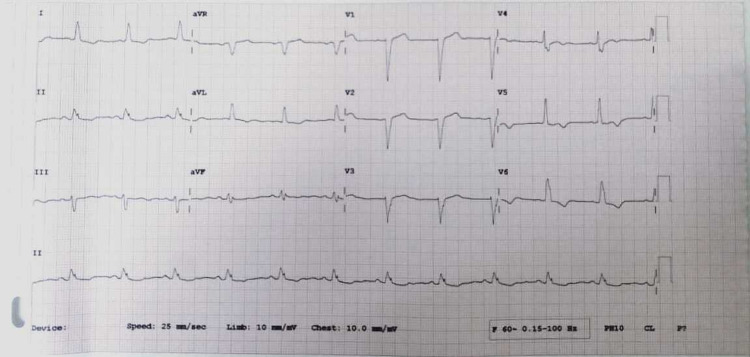
Electrocardiogram showing left bundle branch block

On physical examination, her blood pressure was 107/58 mmHg, pulse 78 beats/min, respiratory rate 18 breaths/min, and temperature 38.2°C. Her systemic examination was unremarkable and her laboratory investigations including full blood count and inflammatory markers (including erythrocyte sedimentation rate and C-reactive protein levels) were within the normal reference range. However, her troponin-I was raised and an electrocardiogram showed a new left bundle branch block (Figure 1). Chest X-ray was also normal.

**Figure 2:**
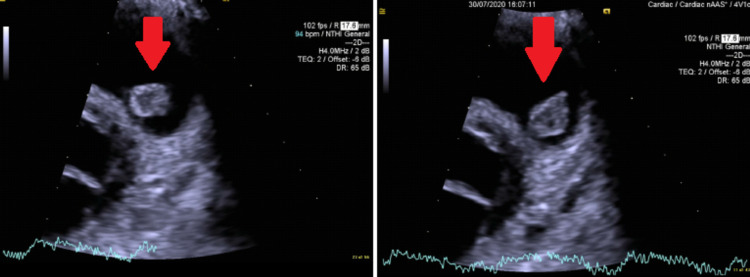
Echocardiogram showing a 1.8 cm vegetation attached to the pulmonary valve

Three blood cultures were drawn with spacing of 30 minutes each and serologies for *Brucella, Bartonella, Coxiella,* and virology were sent, which came back negative. An echocardiogram showed large mobile vegetation of 1.8 cm attached to the pulmonary valve with no valvular destruction, and mild reduction of left ventricular systolic function with an ejection fraction of 50% (Figure 2). We started her on empirical antimicrobial therapy with intravenous gentamicin and vancomycin to treat infective endocarditis. During the first week of antimicrobial treatment, the patient experienced unexplained febrile neutropenia; however, there was no apparent cause.

**Figure 3:**
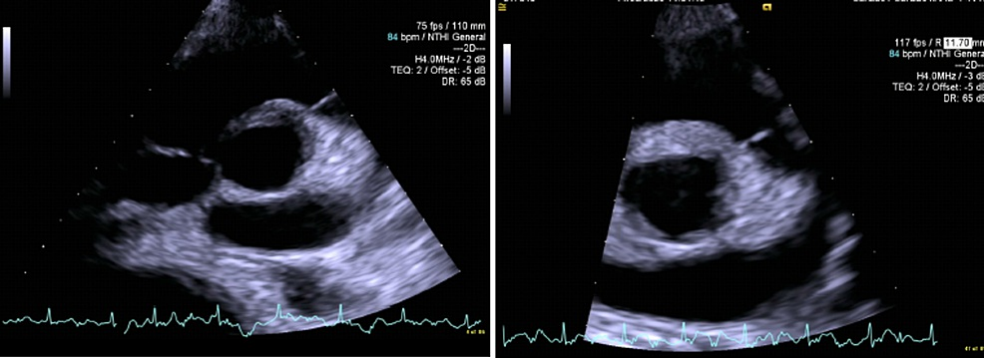
Echocardiogram showing complete resolution of the vegetation after antibiotic therapy

All repeated blood cultures were consistently negative. We consulted Infectious Disease, which advised us to stop vancomycin and to switch to teicoplanin and piperacillin-tazobactam, along with doxycycline. Due to a failure of empiric treatment, we sent the patient's whole blood and plasma for detection of MP by gene amplification via polymerase chain reaction, which came back negative; however, her serology was positive for MP and we started our patient on doxycycline. The patient's fever settled as she improved clinically following the administration of doxycycline. She was discharged to home with doxycycline therapy for a total of two weeks and a repeat echocardiogram at follow-up after two weeks showed complete resolution of the vegetation (Figure 3). 

## Discussion

Clinical presentation of MP varies and most commonly presents as upper and lower respiratory tract symptoms. Though extrapulmonary involvement has been identified, cardiovascular manifestations include pericarditis, myocarditis, congestive cardiac failure, and rarely infective endocarditis [7]. It can affect almost any organ of the body through direct invasion of bacteria or autoimmunity or immune complex deposition [8,9]. Central nervous system manifestations are most noteworthy, especially in children. The presence of extra-pulmonary involvement correlates to a worsened prognosis and can be seen even in the absence of respiratory symptoms [7]. The pathogenesis of endocarditis secondary to MP infection involves the local release of inflammatory cytokines after bacteremia [9]. As cultures are difficult to grow, the diagnosis is based on clinical, radiological, and serological testing [10,11]. The most common organisms involved in culture-negative infective endocarditis include *Coxiella burnetii,* *Bartonella *spp, and *Tropheryma whipplei*, and are associated with a higher mortality rate [12]. Of the organisms that are linked with culture-negative infective endocarditis, *Mycoplasma *spp.-associated endocarditis is most commonly observed in patients with a preexisting cardiac condition [4-6]. 

Our patient presented with fever, raised troponin-I, and a new left bundle branch block on electrocardiography, which gave us a clue that the source of infection is coming from the heart. Failure of empiric antibiotic therapy for infective endocarditis prompted us to look for alternate causes of fever. We started our patient on empirical antibiotic therapy but failed to achieve a response. However, when her serological testing was positive for MP, we switched to doxycycline, after which her symptoms improved.

Our review of the literature revealed limited reports of infectious endocarditis secondary to MP infection. In 1980, Popat et al. described the first case ever of MP causing endocarditis in a young adult [13]. The patient had a history of rheumatic heart disease, who then presenting with fever, lethargy, and malaise was later diagnosed with mycoplasma endocarditis via serology. He was initially started on benzylpenicillin and gentamycin for six weeks, later switched to oxytetracycline, to which he responded very well. Interestingly, his transthoracic echocardiogram (TTE) did not show any vegetations [13]. However, a case reported from Argentina by Scapini et al. showed vegetations in TTE, and the patient was started on clarithromycin after cultures grew MP [14]. In comparison, our patient also had vegetations in his TTE and showed dynamic clinical improvement after initiation of doxycycline, similar to a previously reported case [15].

Through this report, we further strengthen the literature regarding the association of MP with culture-negative infective endocarditis. We believe that robust data are required to determine the true incidence of this association. We provide evidence of the therapeutic success achieved through the doxycycline treatment regimen.

## Conclusions

MP is commonly known as a respiratory pathogen. However, it may involve other systems of the body as well, including the cardiovascular. Rarely, it can cause culture-negative infective endocarditis. We suggest physicians keep a high index of suspicion of MP as a known cause for infective endocarditis in culture-negative cases. 
